# Perceptions and Enhancement Needs of Psychiatric Nurses on Patient Safety Competency: A Phenomenological Study

**DOI:** 10.1155/jonm/3736316

**Published:** 2026-05-08

**Authors:** Chen Shen, Lihua Xu, Qianyun Jiang, Yumei Li

**Affiliations:** ^1^ School of Medicine, Tongji University, Shanghai, China, tongji.edu.cn; ^2^ Psychiatric Department, Shanghai Putuo District Mental Health Center, Shanghai, China; ^3^ Department of Nursing, Shanghai Putuo District Mental Health Center, Shanghai, China; ^4^ Department of Nursing, Shanghai Pulmonary Hospital, Shanghai, China

**Keywords:** patient safety competency, perceptions, psychiatric nurses, qualitative study

## Abstract

**Introduction:**

Patient safety competency among nurses is increasingly emphasized, yet research on patient safety competency among psychiatric nurses remains scarce.

**Aim:**

To deeply explore the cognition of psychiatric nurses on patient safety competency and explore the demands of nurses for enhancing patient safety competency.

**Methods:**

The study was conducted from June to July 2025 with 16 psychiatric nurses. Data were collected through in‐depth personal interviews and thematic analyzed using Colaizzi’s seven‐step phenomenological analysis method.

**Results:**

Three main themes and 13 subthemes are formed: (1) The connotation of patient safety competency, including “protecting one’s own safety is the prerequisite for ensuring patient safety,” “understanding patients,” “observation,” “nurse–patient communication,” “nurse–patient relationship,” and “handling adverse events;” (2) influencing factors of patient safety competency, including “manpower resources,” “leadership,” “teamwork,” and “adverse event management system;” and (3) the demand for improvement of patient safety competency, such as “emphasize theory over practice,” “outdated training content and monotonous formats,” and “desire for more experience sharing.”

**Conclusions:**

This study conducted an in‐depth investigation into the connotation, influencing factors and improvement demands of patient safety competency among nurses in the field of mental health, revealing the specificity of psychiatric nurses’ perceptions of patient safety competency.

**Implications for Practice:**

This provides a basis for health administration departments and hospital managers to develop relevant on‐the‐job training programs and intervention strategies to enhance the patient safety competency of psychiatric nurses, thereby improving the quality of nursing care and delivering superior services to patients. It holds practical significance for the optimization of specialized psychiatric nursing and even the broader healthcare field.

## 1. Introduction

Patient safety is the foremost priority and fundamental requirement in clinical nursing practice, representing the core of nursing quality and a critical focus within healthcare systems globally [1]. Although the global medical technology level has been continuously improving, its development has encountered a bottleneck, and seeking benefits from management has become an inevitable trend. Globally, there are 421 million hospitalizations annually, among which approximately 42.7 million adverse events occur, and the mortality rate due to patient harm is relatively high in countries around the world [2].

Patients with mental disorders often lack insight into their condition, and under the influence of psychiatric symptoms and medications, behaviors such as suicide, self‐harm, violence toward others or property destruction, elopement, choking, and falls pose unpredictable threats to themselves and others, potentially leading to medical disputes [3]. Among adverse events in psychiatric settings, falls are most common (35.70%), followed by medication overdose (14.70%), verbal aggression (12.50%), physical assault (10.70%), and contact with dangerous objects (5.30%) [4]. Insufficient emphasis on patient safety and safety‐focused care may result in temporary or permanent impairment of mental, psychological, and physical functions among psychiatric inpatients [5]. However, psychiatric risks are preventable, and nurses play a crucial role in psychiatric safety [6].

In all aspects of the medical process, nurses should be responsible for the safety of patients. Nurses spend more time with patients than other healthcare professionals, playing a vital role in identifying patient safety risks and ensuring high‐quality care [7, 8]. They actively promote patient safety by meticulously monitoring patient conditions, rapidly identifying risks, and overseeing healthcare processes [9]. Furthermore, nursing activities such as medication administration, infection control, and fall prevention directly impact patient safety [10]. Therefore, maintaining high levels of patient safety competency among nurses is essential for reducing patient safety issues and enhancing the quality of patient care [11].

### 1.1. Background

The patient safety competency refers to the fundamental knowledge, attitudes, and skills that healthcare providers must demonstrate to prevent adverse events and avoid patient harm, thereby serving as a vital component in enhancing both safety outcomes and overall service quality [12]. The Canadian Patient Safety Institute (CPSI) outlined six core domains guiding patient safety practices: safety culture, teamwork, effective communication, risk identification and response, adverse event management, and optimization of personnel and environmental factors, aiming to provide scientific evidence for patient safety education, structuring healthcare professionals’ competencies to better align with patient safety practice needs [13, 14]. Torkaman et al. [15] noted in their study that the patient safety competency of psychiatric nurses serves as a predictor of psychiatric safety care though overall competency levels remain low. A study by Setoodeh et al. [16] in Iran found that there was a significant correlation between compassion competency and patient safety competency in psychiatric nurses.

Currently, there is a lack of qualitative research on psychiatric nurses’ understanding of patient safety competency and the key facilitators or barriers to it. This study qualitatively explores three aspects: (1) the connotation of patient safety competency of psychiatric nurses; (2) promoting and hindering factors of patient safety competency among psychiatric nurses; and (3) the demand for enhancing the patient safety competency of psychiatric nurses.

### 1.2. Theoretical Framework

McClelland [17] conceptualized the “Iceberg Model” of competency was used in this study. Knowledge and skills represent the visible portion of the “iceberg,” manifesting in behaviors that lead to achievements. Beneath the surface lie deeper elements, such as motivation, personal traits, attitudes, values, and self‐concept. In contrast to other domains of public health nursing, safeguarding the safety of psychiatric patients encounters a multitude of barriers. The “Iceberg Model” of competency offers a comprehensive perspective to explore nurses’ perceptions of patient safety competency in the field of mental health. First, clarify the research questions through this theoretical model: What knowledge and skills do psychiatric nurses believe are necessary to ensure patient safety? (surface level/above the iceberg); What are the intrinsic values, self‐perceptions, personal traits, and motivations that drive or influence their practice of these safety behaviors? (deep level/below the iceberg). Based on this theoretical model, the interview outline is designed and the interview process is precisely controlled: the core principle is to move from “visible” to “invisible” and from “concrete” to “abstract.” The interview should follow a natural path of in‐depth exploration, starting from specific and objective facts and behaviors, gradually transitioning to subjective feelings, thoughts, values, and core needs, thereby building trust, reducing defensiveness, and giving respondents the opportunity to organize and reflect on their experiences.

## 2. Methods

### 2.1. Design

This study employed a qualitative descriptive research design. Semistructured interview questions focused on (1) the primary patient safety competency psychiatric nurses need to possess; (2) factors influencing patient safety competency in psychiatric nurses; and (3) strategies for enhancing psychiatric nurses’ patient safety competency.

### 2.2. Study Settings

The data for this study were collected from clinical nurses working in the psychiatric inpatient wards of a mental health center in Putuo District, Shanghai, China. This mental health center, where the first author is affiliated, is a secondary‐level hospital providing both inpatient and outpatient services. The psychiatric inpatient wards operate under a closed‐ward management system, with approximately 540 beds and around 70 clinical nursing staff. The wards are divided into four male wards and two female wards, with a children’s ward currently in the preparatory stage. Admitted patients include those with schizophrenia, bipolar disorder, intellectual disability, and organic brain disorders, with schizophrenia being the predominant diagnosis.

### 2.3. Study Participants

This study is part of a mixed‐methods research design. Prior to the qualitative phase, a cross‐sectional survey was conducted from February to March 2025 among 330 psychiatric nurses selected via convenience sampling from six district‐level mental health centers in Shanghai. The research instruments included (1) a general information questionnaire covering gender, age, marital status, educational background, professional title, position, competency level, years of work experience, employment type, and average number of night shifts per month; (2) the Patient Safety Competency Self‐Evaluation Scale for Nursing Staff; (3) the Psychological Safety Scale; and (4) the Nurse Safety Behavior Questionnaire. The findings revealed that the overall patient safety competency of psychiatric nurses was relatively high, with professional title, competency level, safety behavior, and psychological safety identified as significant influencing factors.

**FIGURE 1:**
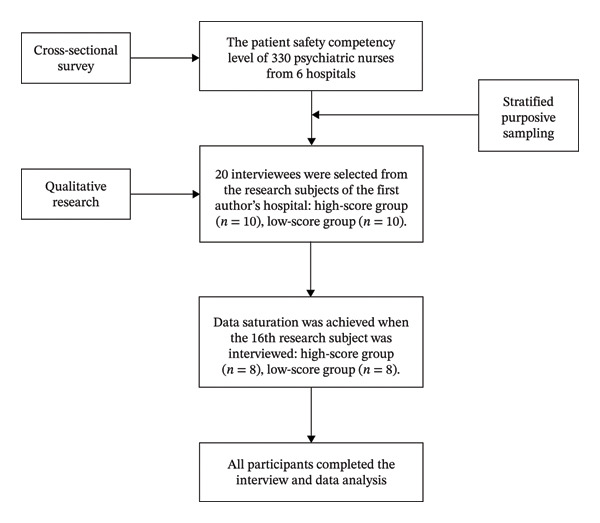
The participant flow diagram.

Based on the findings of the cross‐sectional survey, this study employed a stratified purposive sampling method with equal allocation. The first author personally contacted eligible participants from their affiliated institution, explained the purpose and content of the study, invited them to participate, and obtained their informed consent. Participants were divided into two groups (high and low) based on their patient safety competency scores, aiming to compare experiences and perspectives across different categories, thereby validating and explaining the qualitative implications of the quantitative classification. The inclusion criteria for participants in this qualitative study were the same as those in the quantitative study: participants were required to be registered clinical nurses with at least one year of experience in psychiatric clinical work, and they needed to provide informed consent and voluntarily participate in this study. The participant flow diagram is shown in Figure 1.

### 2.4. Ethical Considerations

This study was approved by the hospital’s Human Ethics Committee.

### 2.5. Data Collection/Procedure

This study employed a descriptive phenomenology design [18]. Data were collected through face‐to‐face, semistructured in‐depth interviews conducted between June and July 2025. Conduct interviews at times convenient for the interviewee and in relatively quiet, private locations. Prior to each interview, participants were informed of the study objectives and relevant considerations, and informed consent was obtained. Conversations were recorded using mobile voice memo applications and transcribed within 24 h postinterview via mobile transcription software. Each interview will last approximately 30–40 min. Upon completion, the content will be summarized on the spot, followed by a brief communication, feedback, and confirmation with the interviewee. Following each interview, a debriefing meeting was conducted to discuss emerging themes and ensure data consistency. During and after the interviews, field notes were recorded to document nonverbal cues and contextual observations; these notes were later reviewed and integrated into the thematic analysis to enrich the interpretation. The interview guide is based on literature review, expert consultation, and the clinical experience of researchers in this field (Table 1).

**TABLE 1:** Interview guide.

1. Please share a few adverse events in psychiatric care that you have personally experienced or are aware of. Which reasons for the occurrence of adverse events do you think are related to nurses? Discuss your perspectives and experiences.
2. Recall instances in your work where adverse events were effectively prevented. How did you achieve this?
3. In psychiatric nursing, under what circumstances do adverse events most likely occur? How can they be effectively prevented and avoided? What details require particular attention?
4. In your view, what is the primary difference between a nurse who effectively ensures patient safety and one prone to errors and incidents?
5. What is your perspective on patient safety? What actions can you take to ensure patient safety?
6. As a nurse, what competencies do you believe are necessary to ensure the safety of psychiatric patients? Which factors do you think will affect these competencies?
7. What shortcomings do you currently recognize in your own psychiatric safety practices? What kind of help is expected to be obtained in response to these shortcomings?

During the interviews, researchers employed guiding and probing techniques [19] to delve deeply into participants’ experiences with patient safety adverse events and their perceptions in patient safety competency. Interviewers maintained the identity of a listener, refraining from judgment to participants’ statements to avoid pressuring or interfering with their free expression of personal views, and control the entire research process according to Streubert’s qualitative research review guidelines [20].

### 2.6. Data Analysis

All written transcripts of individual interviews were coded using NVivo 15.0 software, with data analyzed by two independent researchers. The material was analyzed using Colaizzi’s seven‐step phenomenological method [21]. Next, key information was refined and summarized to identify common characteristics among these statements. Then, themes were formed from the data and linked to the phenomenon under study, while identifying responses that indicate similar perspectives. Finally, the results were returned to the respondents to verify their authenticity. In the event of disagreement during data analysis, we would review the original notes and recordings; should discrepancies persist, respondents would be reinterviewed to resolve inconsistencies until consensus was achieved. This study has some data gaps. The researchers did not perform data interpolation but instead analyzed the reasons for the missing data. For cases with incomplete information, the approach of retaining and using the data and cross‐verifying through triangulation was adopted; for completely invalid data, it was removed and the reasons were explained. Eventually, all core themes reached information saturation, and the absence did not affect the stability of the research conclusion.

### 2.7. Rigor and Reflexivity

The interview was conducted by a postgraduate student of nursing who had received training in interview skills and qualitative methods. Both the interviewer and the interviewees were clinical nurses in the psychiatry department of the same hospital and were acquainted with each other, having a certain degree of mutual understanding. To maintain objectivity and reflexivity and to identify and address potential biases, we employed peer feedback and reflective logs before and after interviews. For instance, inviting a member of the research team who is familiar with qualitative research methods but not directly involved in the project to review the research process, data and preliminary analysis from an external perspective to help identify biases, logical flaws, or limitations in interpretation that the researchers might have overlooked. In addition, throughout the research process, reflective logs were consistently maintained to systematically document procedural details, methodological deviations, and corresponding adjustments at each stage of data collection, coding, and analysis. Concurrently, the potential influence of the researcher’s subjective assumptions and emotional dynamics on the research process was critically examined, and emerging research questions along with their theoretical linkages were promptly identified and clarified.

## 3. Results

Participant characteristics are presented in Table 2, while the 3 themes and 13 subthemes of the study are outlined in Table 3.

**TABLE 2:** Participant characteristics.

ID	Age	Gender	Years of experience	Department	Position	Education	Type of shift	Competency level	Professional title
1	30	Male	10	Male inpatient ward	Nurse	Bachelor’s degree	Shift work	N2	Junior nurse
2	34	Male	12	Male inpatient ward	Nurse	Postgraduate	Shift work	N2	Supervisor nurse
3	26	Female	6	Male inpatient ward	Nurse	Junior college	Shift work	N2	Junior nurse
4	36	Female	16	Male inpatient ward	Nurse	Bachelor’s degree	Day shift	N2	Junior nurse
5	29	Female	9	Male inpatient ward	Nurse	Bachelor’s degree	Shift work	N3	Junior nurse
6	32	Female	11	Male inpatient ward	Nurse	Bachelor’s degree	Shift work	N2	Supervisor nurse
7	28	Female	9	Male inpatient ward	Nurse	Bachelor’s degree	Shift work	N2	Junior nurse
8	38	Female	9	Male inpatient ward	Nurse	Bachelor’s degree	Day shift	N2	Supervisor nurse
9	44	Female	3	Male inpatient ward	Nurse	Bachelor’s degree	Day shift	N2	Supervisor nurse
10	38	Female	8	Male inpatient ward	Nurse	Bachelor’s degree	Shift work	N2	Supervisor nurse
11	34	Female	12	Female inpatient ward	Nurse	Bachelor’s degree	Shift work	N2	Supervisor nurse
12	43	Female	22	Female inpatient ward	Nurse	Bachelor’s degree	Shift work	N2	Supervisor nurse
13	35	Female	10	Female inpatient ward	Nurse	Bachelor’s degree	Day shift	N3	Junior nurse
14	37	Female	9	Male inpatient ward	Head Nurse	Bachelor’s degree	Day shift	N2	Supervisor nurse
15	55	Female	34	Male inpatient ward	Nurse	Junior college	Day shift	N2	Junior nurse
16	39	Female	8	Male inpatient ward	Head Nurse	Bachelor’s degree	Day shift	N2	Supervisor nurse

**TABLE 3:** Three themes and 13 subthemes of psychiatric nurses’ perception of patient safety competency.

Themes	Subthemes	Participants
The connotation of patient safety competency	Protecting one’s own safety is the prerequisite for ensuring patient safety	P1, P9, P13, P14
	Understand the patient	P5, P7, P9, P11
	Observation	P2, P3, P4, P7, P8, P9, P10, P12, P13, P16
	Nurse–patient communication	P6, P9, P10, P11, P12
	Nurse–patient relationship	P6, P8, P12, P13
	Handling adverse events	P9, P11, P12

Influencing factors of patient safety competency	Manpower resources	P12, P13, P15
	Teamwork	P2, P5, P11, P16
	Leadership	P7, P8, P12, P14
	Adverse event management system	P4, P5, P6, P7, P10

The demand for improvement of patient safety competency	Emphasize theory over practice	P4, P10, P11
	Outdated training content and monotonous formats	P7, P14, P16
	Desire for more experience sharing	P1, P3, P14

### 3.1. Theme 1. The Connotation of Patient Safety Competency

#### 3.1.1. Subtheme 1.1. Protecting One’s Own Safety Is a Prerequisite for Ensuring Patient Safety

Participants emphasized that while ensuring patient safety is paramount, it must be predicated on safeguarding one’s own safety. They reported that when managing agitated patients, personal protection measures must be implemented promptly and effectively to protect both patients and nurses.

• My entire focus is preventing incidents involving patients. During my shift, patient safety is the absolute priority—but only after ensuring our own safety as nurses. That is my fundamental stance. Other minor details might not receive as much attention. (P1)
• For newly admitted patients who become emotionally agitated and may attack us, we must prioritize both the patient’s safety and our own as healthcare providers. Timely protective restraints must be applied. (P14)
• While ensuring patient safety, we must also safeguard our own security. (P9)
• When dealing with agitated patients, prioritize your own safety and implement protective measures. If you cannot even ensure your own safety, how can you talk about patient safety? (P13)

#### 3.1.2. Subtheme 1.2. Understanding Patients

Participants described that understanding patients enables better observation. They discussed how nurses’ varying levels of familiarity with new versus established patients affect their attentiveness and vigilance. Additionally, participants mentioned that each patient’s condition is unique and that nurses must maintain a comprehensive, in‐depth, and dynamic understanding of the patient’s status rather than relying solely on assessment tool scores to determine risk levels.

• Some nurses lack comprehensive understanding of conditions and are unclear about patients’ specific manifestations. They have relatively limited clinical experience and insufficient foundational knowledge. (P5)
• If I’m not particularly familiar with a patient—if I do not know their habits—my observations would not be as thorough. For instance, with many long‐term patients, I will note what tends to happen during other shifts. Once I know that, I pay extra attention to that patient. (P7)
• Over time, or after consistently caring for certain patients, one might become lax and less vigilant. But with a new patient, especially someone admitted for the first time, you heighten your alertness because you do not know them and cannot predict their behavior. (P9)
• Based on each patient’s unique circumstances, we should also assess aspects beyond the evaluation form. For example, even if a patient is classified as low risk on the form, they might appear dangerous. Drawing on experience, you might anticipate they could fall at night. You need to be aware of this and conduct targeted, more frequent rounds. (P11)

#### 3.1.3. Subtheme 1.3. Observation

Participants emphasized that observing patients is the most critical means for preventing adverse events. They noted that psychiatric patients often struggle with self‐expression and communication, requiring nurses to conduct meticulous, dynamic observations to promptly detect changes in condition or behavioral signs, enabling timely intervention.

• The primary method for preventing adverse events is monitoring patients’ conditions. Yes, it is crucial to promptly detect changes in their condition, communicate immediately with doctors, adjust medication as needed, and most importantly, ensure thorough handover during shift changes. (P12)
• Some patients may conceal their thoughts. Even when asked, they might not disclose their current symptoms. These require your observation, subtle observation. (P13)
• Additionally, some patients exhibit warning signs before impulsive episodes, they might bang on windows, knock on doors, or become verbally abusive. You sense something is off, especially if the patient has been stable otherwise. Should not you then place them in an observation room? (P10)
• We must also pay attention during patient‐family calls. What exactly did the patient discuss with their family? After each call, the patient’s mood may shift. Following key events like visiting hours, patients are particularly prone to emotional changes. (P3)
• Some patients hide things you had never notice. One patient taking risperidone oral dissolving film would stick the medication to his tongue and then attach it to the rim of his cup while drinking water. He had cover the cup, so it stuck to the lid. Even when he opened the cup, you would not see it. (P2)

Interviewees emphasized that nurses’ grasp of basic medical knowledge is crucial for observing and assessing patients’ physical illnesses. As psychiatric patients age, the prevalence of physical diseases increases. However, psychiatric nurses often lack foundational knowledge in internal and surgical medicine, hindering their ability to promptly identify and evaluate patients’ physical conditions, resulting in some patients being unable to receive effective treatment.

• If you do not have some basic medical knowledge, you would not be able to detect certain illnesses in patients. When a patient says they feel unwell, you would not be able to determine if they are actually sick, nor will you take it seriously. (P7)
• Yes, it is true that when patients present with internal or surgical issues, we cannot make a diagnosis. Moreover, there are numerous physical complications or adverse reactions caused by the antipsychotic medications used in psychiatric patients. But while we might notice they do not seem well, we cannot actually pinpoint what is wrong. Right, some symptoms are actually quite obvious but because we lack the knowledge, we cannot make a proper assessment. Yes, this includes when outside doctors come for consultations and we listen in—we always feel like we do not understand anything. (P3)
• Moreover, psychiatry is not solely about mental disorders. With the aging of many psychiatric patients, their physical illnesses have increased. Actually, learning some internal medicine and surgery knowledge is beneficial for your work. Even though we are in a specialized hospital now, I feel it is particularly like internal medicine—studying it is genuinely helpful. Take a patient we had today, he was sitting there perfectly fine, but when I saw him shiver twice, something felt off. I went in to take his temperature, and sure enough, he had a fever. (P8)
• Because even though we are dealing with psychiatric patients, as they age, they may develop various somatic conditions related to internal or surgical medicine. Therefore, in their daily observations and interventions, nurses must also possess some knowledge of internal and surgical medicine. Only then can they promptly identify and address emerging somatic issues in patients. (P9)

Participants also discussed potential safety hazards in the environment and facilities. They emphasized that during rounds, nurses must not only observe patients but also monitor the environment and facilities, promptly identifying, reporting, and addressing potential hazards to eliminate safety risks.

• Last time we discovered a drain cover that was very thin, like a razor blade, extremely sharp. A patient could use it to harm themselves or others. We promptly reported it to the General Affairs Department, and the drain cover was replaced, eliminating a safety hazard. (P7)
• For instance, with this patient who eloped, if we had conducted timely and thorough rounds, or routinely inspected the safety of surrounding hardware facilities, we could have detected, investigated, and addressed the issue promptly, thereby reducing the risk of elopement. (P9)
• First, our rounds for patients under protective restraints were inadequate. Additionally, since I was new at the time, I did not pay enough attention to the overall environment. Later, when I transferred to another unit, I noticed they documented potential hazards, likely because they had already conducted thorough environmental inspections. (P16)
• During ward rounds, observe not only patient conditions but also the surrounding environment, such as check for water on the floor, damaged windows, etc. Address issues immediately, report repairs promptly, and eliminate safety hazards. (P4)

#### 3.1.4. Subtheme 1.4. Nurse–Patient Communication

Participants emphasized that maintaining timely and effective communication with patients is crucial for understanding their thoughts, identifying discomfort, and calming their emotions. Some participants reported that when interacting with patients, one must be mindful of approach and method, tailoring communication styles to different individuals.

• When implementing protective restraints, you must explain to the patient: “You are just feeling overly agitated right now, so I need to restrain you temporarily to prevent you from harming others or yourself.” I believe this explanation is essential; otherwise, once the restraints are removed, the patient might develop delusions about you. (P10)
• We likely did not communicate with him enough beforehand and failed to monitor his psychological changes. No one wants to be hospitalized, especially someone as young as him. After admission, we did not engage with him promptly enough. By missing his psychological shifts, we may have caused communication delays that allowed certain thoughts to take root. (P12)
• It is difficult for us to detect when a patient has caught a cold or feels unwell these days. With so many patients to monitor individually, it is impossible to observe everyone meticulously. We rely on patients reporting their discomfort; identifying it ourselves is challenging. (P11)
• I truly believe the approach when interacting with patients is crucial, really crucial. Even the tone of your voice matters sometimes, right? With some patients, you can be a bit harsher in your words, but not with others. But that does not mean we should just placate them; I still think we need to be mindful of our methods. (P10)

Participants noted that some family members are unreasonable and difficult to communicate with. To avoid medical disputes, they avoid interacting with families, creating tension in the nurse–patient relationship. Some participants, however, stated that good communication with families can strengthen the nurse–patient bond, potentially preventing disputes. Some participants pointed out that families often know patients better than medical staff because patients may be more willing to communicate with them, making families a crucial source of patient information.

• I tell the family, “This patient eats very quickly. Last time he choked on his food. Why do not you stay a little longer? Take some time to sit with him, talk to him, and wait until he finishes eating before leaving.” If you directly tell the family what they cannot bring, they would not listen to you. If you yell at them a couple of times, I am telling you, they will go file a complaint against you. (P6)
• We rarely communicate with family members except during patient admission/discharge or visiting hours. Unless they approach us or there is an emergency, we do not initiate contact. Typically, the condition is conveyed by the doctor. (P10)
• If you help the family out with things during normal times, chat with them when there is nothing going on, and build a good rapport, then when something really happens—nothing too serious—the family usually would not hold it against you. Like we have a patient here who frequently has seizures and falls. The family could blame you, or they might not. But since we have always had good conversations with them, the family does not really care. (P9)
• Some patients may distrust us. We can communicate by consulting their families. Some patients report fewer symptoms; when they feel unwell, they tell their families but not us. Some families then relay this information to us. (P11)

#### 3.1.5. Subtheme 1.5. Nurse–Patient Relationship

Participants believe that to ensure ward safety, different approaches and attitudes should be adopted for different patients. For certain patients, it is necessary to be strict with them and have a tough attitude, which will be more conducive to the management of the department. Conversely, with stable, lucid patients, fostering positive relationships and involving them in ward management enhances safety. Some participants emphasized maintaining appropriate distance from all patients, heighten safety awareness, and eliminate potential risks.

• Different patients require different methods and attitudes. Some say you should not get too close to patients, but we should build good relationships with cooperative ones. Plus, cooperative patients can help monitor and manage others. (P13)
• With certain patients, you need to be firm because many respond better to strictness than gentleness. I find that if you take a tougher stance, they might become a bit wary of you, which can help them behave more appropriately at night. (P12)
• Like when I am on duty, if a patient falls, some will run over to tell me—I rush to check on them, right? You really need to build good relationships with stable, mentally alert patients. They will pay extra attention and help you out during your shift, right? (P8)
• Do not get too close to patients. If you become too friendly, you will gradually become lax. If you feel sorry for them and help them, your safety awareness will fade. By keeping your distance, your safety awareness strengthens, and you enforce protocols strictly. If you are close, you might turn a blind eye to certain things, creating safety hazards. (P6)

#### 3.1.6. Subtheme 1.6. Handling Adverse Events

Participants reported that certain adverse events in psychiatric settings are unavoidable, making emergency response capabilities of nurses particularly crucial. Identifying adverse events in a timely manner and handling them appropriately to minimize losses and damages is an essential competency that every psychiatric nurse should possess.

• Adverse events are inevitable in psychiatric settings. The ability to promptly identify and effectively manage these events to minimize losses and harm is essential for every psychiatric nurse. (P11)
• Emergency response capability is also vital. During emergencies, knowing what actions to take and what to avoid, distinguishing between critical and noncritical matters, and prioritizing tasks are essential. Address critical issues first, then noncritical ones, and never avoid the serious to focus on the trivial. (P9)
• Ultimately, the nurse’s adaptability determines the outcome of an emergency. Effective handling can minimize a major incident, while poor management can escalate a minor issue. (P12)

### 3.2. Theme 2. Influencing Factors of Patient Safety Competency

#### 3.2.1. Subtheme 2.1. Manpower Resources

Participants indicated that compared with the day shift, there were insufficient nursing staff on the middle and night shifts, and there were more uncontrollable factors, making it impossible to fully ensure patient safety. They suggested addressing nursing shortages through rational scheduling, increasing staffing reserves, or expanding the number of handymen.

• After all, with only one person on the late‐night shift, you have to watch both the front and the back—it is impossible to keep an eye on everything. If we nurses spot issues promptly, our emergency response capabilities are still adequate. Moving forward, we should strengthen staffing reserves and adjust the scheduling. (P13)
• During the night shift, understaffing can lead to adverse nursing events. Day shifts are somewhat better because there are more staff, but with fewer people, it is impossible to patrol 24/7. At most, we can patrol at set intervals, and even then, we might not catch every issue. For instance, some patients might fall right after the nurse leaves their room. You cannot blame the nurse, as they have already followed protocol by patrolling every hour. (P15)
• If we cannot assign nurses, we will assign student workers instead. Student workers can assist and help in emergencies, whereas having just one person on the night shift is too thinly stretched. (P12)

#### 3.2.2. Subtheme 2.2. Leadership

Participants reported that the extent to which leadership prioritize and support patient safety significantly influences the safety awareness of the staff. They proposed that head nurses should take the lead in ensuring patient safety, setting an example by their own actions, thereby instilling confidence in the entire team and preventing adverse events.

• Every leader has different priorities. Some focus more on safety, while others emphasize research—everyone has different perspectives. You might feel you have ensured safety, but it does not necessarily get recognized. (P14)
• If leadership hears your concerns but fails to take action, offer support, or implement improvements, does not that create new safety hazards? (P7)
• If you observe issues or hazards within the department and report them to leadership and they support you and implement immediate improvements, that significantly enhances ward safety. (P8)
• If the team, under the head nurse’s leadership, has clear objectives and well‐defined responsibilities, and everyone follows established protocols and workflows, I believe we can be reasonably confident in preventing adverse events to a certain extent. (P12)

#### 3.2.3. Subtheme 2.3. Teamwork

Participants emphasized that ensuring patient safety is a collective effort that extends beyond nursing staff, requiring the active participation and collaboration of all relevant departments. Participants acknowledged that patient safety is not the responsibility of any single nurse but of the entire nursing team. Only through seamless cooperation among the entire nursing team can the long‐term stability and safety of the entire department be effectively guaranteed.

• Ensuring patient safety is not solely our nurses’ responsibility; it requires joint efforts from doctors and other support departments like housekeeping, lab, pharmacy, and rehabilitation. This mutual cooperation enables timely and efficient handling of any patient situations that arise. (P2)
• Safety is a team responsibility. We morning shift nurses admit patients, right? The third shift must be briefed on their meals, medication intake, psychological state, and behavior. I alone cannot guarantee patient safety. I can only ensure timely intervention if their condition changes during my shift and conduct thorough handovers to prevent similar incidents, correct? (P11)
• Teamwork is crucial. Every task is interconnected—no one can adopt a “not my problem” attitude. We have shift handovers, and if a patient shows early warning signs during one shift—like medication reactions or unusual movements—attentive nurses document these observations. This allows the next shift to prioritize monitoring that patient. (P16)
• Moreover, some doctors do not grasp patients’ conditions as concretely as we do. When a patient is admitted, it is like a new admission—the medical history might state they were violent at home. But after admission, we observe the patient is quite stable. Family members might report poor sleep at home, yet we see them sleeping soundly through the night. So, you need to promptly relay this feedback to the doctor to adjust the treatment plan. (P5)

#### 3.2.4. Subtheme 2.4. Adverse Event Management System

Participants indicated that the adverse event reporting process and subsequent corrective actions are cumbersome, leading to reluctance to report incidents, which results in many adverse events not being taken seriously or rectified. Participants candidly stated that approach of leadership to assigning responsibility and implementing rewards/punishments for adverse events is problematic, negatively impacting everyone’s morale and attitude.

• I feel many adverse events are handled internally, just resolved within our own department. For less serious incidents, people try not to report them because once reported, you have to implement corrective actions and write all sorts of corrective measures. After reporting, the analysis usually ends up placing the blame on us nurses, so people tend to avoid reporting certain things. In this situation, issues go unnoticed and remain unresolved. (P4)
• Why does not anyone want to report adverse events? Besides the hassle of writing corrective actions, it might also be that if you report it, no one acknowledges your efforts. So why bother? If I report it and get no recognition, and people even mock me, why should I report it? Right? (P5)
• According to our hospital’s policy, incidents occurring during the morning shift are solely the responsibility of the morning shift. Because several adverse events in our department were reported with the morning shift nurse listed as responsible. This responsibility should be shared by the department, not borne by one person. Only then will everyone develop a safety consciousness. (P10)
• When accidents like this happen, the nurses are actually the most upset. Because if it happened on our shift, we are the ones directly involved. Of course, no one wants accidents to happen. But some things are unavoidable. The only question is whether we handled the aftermath properly. Yes, if we handled it well, I think the Nursing Department could acknowledge our strengths. If we did not handle it well, you can point out exactly what we did wrong and how we should handle it next time. Share your insights with us, do not just criticize, right? Teach us what you handled well, what could be improved, and how to do better next time. That way, we know how to perform better in the future, right? (P7)
• Fear of punishment makes us constantly anxious at work. Yet, the more we dread mistakes, the more likely they become, because tension clouds our judgment and affects our state of mind, right? When we feel more relaxed and less pressured, our work performance naturally improves. (P6)

### 3.3. Theme 3. The Demand for Enhancing Patient Safety Competency

#### 3.3.1. Subtheme 3.1. Emphasize Theory Over Practice

Participants noted that patient safety training overemphasizes theoretical learning while neglecting practical drills, preventing them from applying what they learn.

• Let me put it this way: memorizing theory is useless without real‐world application. Practical training is not about reciting procedures, it is about actually doing them. Our so‐called fall prevention protocols and fire response procedures are meaningless. When incidents actually occur, they fail. Then we must ask: why? We drill year after year, month after month, until everyone’s brain is fried. (P4)
• But the problem is that textbook knowledge differs from clinical practice. You could memorize an entire book, pass nursing department quizzes flawlessly, yet real clinical application is entirely different. Only after experiencing incidents on your shift do you truly understand that it takes time to accumulate that knowledge. (P11)
• What good is glancing at your phone or the Nursing Assistant app? Right, hands‐on practice, on‐site training, we just do not have the time. Exactly, so this is useless to me, it is all just theoretical talk. (P10)

#### 3.3.2. Subtheme 3.2. Outdated Training Content and Monotonous Formats

Participants indicated that patient safety training content was outdated and monotonous, diminishing their motivation and interest in learning. They expressed a desire to access the latest research developments in psychiatry to enhance both their clinical practice and research capabilities.

• New nurses definitely care about these things. But when it is the same format over and over, everyone just gets numb. (P14)
• After working for so long, I have lost count of how many trainings I have attended. You can recite the entire process by heart, right? You just become numb to it and stop paying attention. (P16)
• I believe continuous learning and professional development are essential at any stage. With advances in modern science, we must stay updated on psychiatric developments and enhance our research capabilities. This includes studying the latest psychiatric advancements, constantly improving ourselves, and pursuing ongoing learning and progress. (P7)

#### 3.3.3. Subtheme 3.3. Desire for More Experience Sharing

Participants suggested hospitals should organize more exchange activities focused on patient safety practices, where senior nurses with extensive experience can share and impart their expertise.

• The hospital could have senior nurses conduct lectures or hold regular seminars to share their practical experiences with us, as some things simply cannot be learned from textbooks. (P1)
• Our training may emphasize being kind to patients and avoiding provocative language. But how to phrase things, such as the specific communication techniques, requires personal practice. For nurses who grasp concepts more slowly, I think a senior nurse or one with strong problem‐handling skills should teach them communication strategies or how to address specific issues. (P14)
• When I first joined the hospital, I listened to many senior nurses share their unique experiences, like precautions during night rounds. Communicate frequently with more experienced nurses to learn from their expertise. Always ask questions when uncertain. If something is unclear, make sure you fully understand it before proceeding. (P3)

## 4. Discussion

Previous studies on patient safety competency have mostly been quantitative research based on questionnaires, and there is still a lack of objective and comprehensive qualitative research on nurses’ patient safety competency. This study, for the first time, takes a qualitative approach to explore the perceptions of psychiatric nurses regarding patient safety competency.

In the following discussion, we will focus on elaborating the differences or unexplored aspects between the perceptions of patient safety competency among psychiatric nurses perceptions in this study and previous research on patient safety competency of nursing staff from three perspectives: the connotation, influencing factors, and improvement needs of patient safety competency, in order to reflect the specificity of psychiatric nurses’ perceptions on patient safety competency.

### 4.1. Connotations

Psychiatric nurses in this study prioritized their own safety over patient safety. This attitude seems to be inconsistent with the patient‐centered concept proposed in the patient safety competency framework put forward by CPSI. A likely reason is that individuals with mental disorders exhibit significantly higher rates of violent attacks and criminal behavior compared to the general population [22], and their violent acts are more sudden and destructive [23], posing severe threats to the safety of psychiatric healthcare providers. This necessitates psychiatric nurses possessing stronger self‐protection awareness than nurses in other departments. Balancing patient safety with personal safety demands constant reflection and practice from every psychiatric nurse in their daily work.

Nurses’ keen observation is crucial for promptly detecting changes in condition and preventing errors or accidents [24]. Our research has found that the assessment of psychiatric patients’ clinical conditions, behaviors, emotional changes, and medication compliance and the identification of potential safety risks in the environment largely rely on the nurses’ observational skills. Psychiatric patients, particularly those lacking insight, experiencing disorganized thinking, or suffering from severe somatic diseases, often cannot proactively or accurately articulate their discomfort or needs [25]. Close monitoring and focused observation of high‐risk patients are vital steps in identifying and eliminating potential safety hazards. The ward environment itself serves as a safe haven [26], and psychiatric nurses are pivotal in creating this secure setting [27]. By conducting regular, meticulous environmental safety inspections and reviewing patients’ belongings within ethical and regulatory frameworks, nurses physically eliminate hazards, fostering a genuinely safe therapeutic environment.

The nurse–patient relationship is widely acknowledged as a foundational therapeutic modality, occupying a central position within psychiatric nursing practice [28]. A positive nurse–patient relationship fosters a therapeutic environment characterized by understanding, respect, and care, which encourages patients to express their inner distress and conflicts verbally rather than resorting to risky behaviors. This significantly enhances patient safety at a fundamental level. However, nurses face challenges in setting and maintaining boundaries in the nurse–patient relationship [29, 30]. Some of the recipients in this study expressed understanding for the nurses’ efforts to maintain friendly relationships with patients for the safety of the department; while others were concerned that overly close friendly relationships between nurses and patients might lead to boundary violations. The reason for this situation might be the lack of understanding of the standards and policies regarding professional relationships and boundaries for nurses. Nurse managers should provide a platform for thinking and inquiring about potential conflicts or boundary crossings and discuss and explain the standards and policies regarding professional relationships and boundaries [31, 32], filling the gap in nurses’ understanding of this area.

### 4.2. Influencing Factors

Nurses are of vital importance in patient care, and having an appropriate level of nurse staffing is also extremely crucial for patient safety. Hanrahan et al. [33] pointed out that having an adequate number of psychiatric nurses in the hospital is undoubtedly a key component of high‐quality inpatient psychiatric care. Cho et al. [34] found that the number of patients each nurse was responsible for and whether they worked overtime were closely related to the reporting of patient safety conditions, the quality of care, and the probability of not completing care tasks due to insufficient time. However, in actual practice, it is quite challenging to properly allocate nursing staff, as various factors such as the individual characteristics of the nurses, the features of the working environment, and the characteristics of the patients, as well as the outcomes for both the nurses and the patients, must all be taken into account [35]. How to make reasonable allocation of limited human resources and fully ensure the safety of psychiatric patients have posed a challenge for nursing managers.

Hospital leaders exert the most fundamental and far‐reaching influence on the development of organizational safety culture and climate [36]. The relationship between leadership and safety plays a crucial role in creating positive safety outcomes for patient care [37]. Xie et al. [38] developed a patient safety leadership program which, through training for nurse managers, significantly improved not only their leadership behaviors but also positively impacted the safety behaviors of clinical nurses. Boamah et al. [39] noted in their research that transformational leadership has a significant positive impact on workplace empowerment, which in turn enhances nurses’ job satisfaction and reduces the frequency of adverse patient events. To cultivate a better work environment, healthcare administrators must prioritize policy reforms that foster leadership development and mentorship for both current and future managers.

The adverse event management system promotes a just culture by distinguishing between human error, reckless behavior, and flawed systems. It encourages nonpunitive reporting, enabling healthcare workers to voluntarily report adverse events, thereby shifting the focus from attributing individual culpability to identifying latent systemic failures, and fundamentally preventing similar incidents from recurring. In contrast, by treating medical errors primarily as individual failings, the traditional blame culture discourages transparency and obstructs the identification and resolution of systemic issues [40]. Consequently, healthcare institutions should cultivate a constructive safety culture and a fair environment by explicitly defining which behaviors constitute acceptable, system‐related oversights and which constitute reckless or intentional actions warranting individual accountability. This clarity ensures that staff perceive open reporting as a secure and valued practice, confident that system‐related disclosures will not result in unjust disciplinary measures.

### 4.3. Enhancing Demand

As far as we know, this study is the first to explore the demand for enhancing patient safety competency of nurses from a qualitative perspective. The interviewees in this study expressed a strong need to enhance their competency in patient safety, hoping to acquire the most up‐to‐date and practical knowledge and skills for ensuring patient safety through more diverse channels and in more innovative formats. Based on the identified need to enhance patient safety competency among interviewees in this study, it is recommended that healthcare institutions and nursing management implement extended and more intensive pre‐employment training programs. These should emphasize simulation‐based exercises focused on risk assessment, de‐escalation strategies, and restraint procedures. Additionally, regular safety case conferences should be institutionalized, enabling teams to collaboratively analyze recent or near‐miss incidents and disseminate lessons learned.

## 5. Limitations

Due to the limitations of conditions, this study only selected psychiatric nurses from the institution where the first author is located as samples, which may not be representative of a broader group of psychiatric nurses. Additionally, during the interviews, due to the sensitive nature of some questions, the respondents were concerned that their career prospects might be affected. As a result, they did not fully express their true thoughts or held back in their responses. During the data collection stage of qualitative research, it is extremely vulnerable to the influence of response bias [41, 42]. Social desirability bias, as a common type of response bias, refers to the tendency of research participants to describe themselves in positive terms in order to shape a self‐image that conforms to social expectations or certain situations, rather than answering truthfully and accurately [43]. Respondents in this study may have been influenced by social desirability bias, potentially undermining the credibility and authenticity of the research. Furthermore, critical themes may have been deliberately concealed, preventing the full scope of issues from being explored and resulting in a one‐sided analytical perspective.

## 6. Conclusion

This study provides an in‐depth analysis of the largely overlooked patient safety competency among psychiatric nurses, offering a qualitative exploration of their understanding of this competency, including its connotations, influencing factors, and the need for enhancement. Future research efforts should focus on the following aspects: (1) Further explore the connotation of the patient safety competency of psychiatric nurses. (2) Through quantitative research, explore the influencing factors of the patient safety competency of psychiatric nurses. (3) Probe into the improvement plans for the patient safety competency of psychiatric nurses.

## 7. Implications of the Study

The theoretical significance of this study lies in providing a basis for health management departments and hospital administrators to develop relevant on‐the‐job training programs and intervention strategies aimed at enhancing the patient safety competency of psychiatric nurses. Its practical value is reflected in contributing to the improvement of psychiatric nursing management, thereby elevating the overall quality of nursing care and delivering high‐quality services to psychiatric patients. This holds practical relevance for the advancement of specialized psychiatric nursing and the optimization of the broader healthcare system.

## Author Contributions

The research concept was collectively proposed by all authors. The principal investigator of this study is Chen Shen, who was responsible for all interview tasks. Chen Shen and Lihua Xu were accountable for material preparation and data collection. Chen Shen, Lihua Xu, and Qianyun Jiang were involved in the analysis work. Shen Chen drafted the initial version of the paper, which was subsequently refined by Lihua Xu, Yumei Li, and Qianyun Jiang. All authors reviewed the manuscript prior to submission and made contributions.

## Funding

This study was supported by the 2024 Hospital‐level Scientific Research Project of Shanghai Putuo Mental Health Center (PTJW202503).

## Ethics Statement

Ethical approval for this study was obtained from the Shanghai Putuo Mental Health Center Ethics Committee (no.: 001) on 23.12.2024.

## Conflicts of Interest

The authors declare no conflicts of interest.

## Data Availability

Due to ethical concerns and issues related to the personal privacy of the respondents, access to the data is restricted.
